# Simulating data breaches: Synthetic datasets for depicting personally identifiable information through scenario-based breaches

**DOI:** 10.1016/j.dib.2024.111207

**Published:** 2024-12-06

**Authors:** Abhishek Sharma, May Bantan

**Affiliations:** a Indian Institute of Technology Madras, India; b Kennesaw State University, United States

**Keywords:** Synthetic data generation, Data breach, Personally Identifiable Information (PIIs), Data classes, Hacked data dumps

## Abstract

With hackers relentlessly disrupting cyberspace and the day-to-day operations of organizations worldwide, there are also concerns related to Personally Identifiable Information (PII). Due to the data breaches and the data getting dumped on the clear web or the dark web, there are serious concerns about how the different threat actors worldwide can misuse the data. Also, it raises the question of how hackers can create a profile of an individual starting from one data leak and getting more details on individuals with the help of Open Source Intelligence (OSINT). Furthermore, there is a dilemma in utilizing data breach datasets dumped on the clear web or the dark web because of the sensitive nature of the information. There can be issues related to ethics, law enforcement, and legal use of data. Thus, to tackle this, we will construct synthetic datasets that will allow researchers and professionals to understand how data leaks can be dangerous and how hackers can connect the dots further by creating complete profiles of individuals. We have programmatically generated a synthetic master record of 4 million unique individuals with complete profiles of their PIIs, and then using the master record, we have further generated 16 scenario-based datasets by creating a fictitious narrative of data breaches covering different industry types. These datasets will facilitate researchers and industry professionals in understanding the distribution of PIIs across data breaches. The data classes represent the nature of PIIs sourced from ‘Have I Been Pwned?’ to create synthetic records. The synthetically generated records are shared with the code in this paper to facilitate future researchers and practitioners to generate customized synthetic records according to their requirements, enabling transparency in terms of reusability, reproducibility, and replicability.

Specifications Table

**Table:** 

Subject	Data Engineering
Specific subject area	Synthetic Data, Data Breach, Personally Identifiable Information (PII)
Type of data	Master data record (full profile of PIIs synthetically generated) 4 million records (.csv format file). Furthermore, 16 datasets representing different data breach scenarios with different numbers of records are derived from the master data record (.csv format file). All the files are stored in a .csv file format. The python code is saved in an .ipynb format, which is a python notebook.
How the data were acquired	Data Classes were taken from real-life data breach incidents, which were sourced from ‘Have I Been Pwned?’ (HIBP). Data Classes refer to PIIs that are compromised or exposed in a data breach, such as email addresses, passwords, usernames, IP addresses, phone numbers, and credit card information. The synthetic data records involving PII were generated using the Faker python library. The passwords were sourced from ‘rockyou.txt’ password list. The city, state and zip code was assigned randomly to the records through the ‘pyzipcode’ python library. The US area codes was taken from North American Numbering Plan Administrator (NANPA).
Data source location	The raw list of data breaches and its data classes is available at: https://haveibeenpwned.com/ The passwords list is available through Kali Linux operating system (OS) distribution (https://www.kali.org/) and the wordlists package for the OS (https://www.kali.org/tools/wordlists/) and the package tracker website (https://pkg.kali.org/pkg/wordlists) The US Area Codes are sourced from North American Numbering Plan Administrator (NANPA): https://nationalnanpa.com/
Data accessibility	Repository name: Mendeley Data Data identification number: 10.17632/sxfjgcynjv.2 Direct URL to data: https://data.mendeley.com/datasets/sxfjgcynjv/2

## 1 Value of the Data

• The datasets consist of synthetically generated data records consisting of classes representing the nature of PIIs taken from real-life data dumps.
• The generated synthetic datasets will help map the relationships between the data. Researchers and industry professionals can analyze the data to trace patterns and relationships between the datasets representing different data breaches. Open Source Intelligence (OSINT), Privacy-Preserving machine learning, and other analytical techniques can be used on the dataset to gain more insights.
• The use of synthetic datasets in data breaches can be useful in ethically analyzing the data, where researchers and industry professionals need not worry about sensitive real-life information.
• These programmatically generated synthetic datasets aim to enhance transparency in terms of reusability, reproducibility, and replicability. The code for synthetic data generation for the datasets (master record and scenario-based datasets) is provided with the paper.
• This dataset provides a baseline for future research directions. The master record can be further used to create more scenario-based datasets. The master record will be helpful in a case where researchers and industry professionals want to create new records based on their needs (the code is also provided to ensure reusability, reproducibility, and replicability). The code can be modified to create more dataset variations distributed across various regions as well.

## 2 Background

Data breaches have become a significant threat to both individuals and organizations in todayʼs digital age. Breaches often lead to identity theft, where sensitive PIIs such as Social Security numbers, credit card details, and addresses are exploited by threat adversaries, resulting in significant financial losses [4,5]. Additionally, the reputational damage that follows such breaches is profound. The Ashley Madison leak in 2015 exposed the personal data of over 30 million users, leading to significant emotional distress, public shaming, and relationship breakdowns for those involved. The breach highlighted major failures in data security. It eroded trust in online privacy as many individuals faced legal, financial, and psychological consequences due to their data being made public [6,7]. With the help of several sophisticated scripts, tools, and mechanisms available online, the threat adversaries can also create a profile on individuals by connecting the dots between different data breaches through Open Source Intelligence (OSINT) [[8], [9], [10], [11]]. This can further enable the hackers to explore more sensitive information about the individuals. From the perspective of researchers and practitioners, one of the main concerns regarding analyzing data breaches is the sensitive nature of information [[12], [13], [14]]. Investigating and analyzing these breaches often requires access to compromised data, which poses significant ethical concerns related to privacy and consent, as real-world datasets are often highly sensitive and include personal information that cannot be shared or analyzed ethically. This makes gaining insight into adversaries’ methods and understanding the full extent of breaches complex [4,[15], [16], [17],^19^]. Synthetic data are artificially generated datasets that mimic real dataʼs statistical properties without containing any sensitive information [[18], [19], [20]]. Based on this, our objective is to generate a synthetic dataset for scenario-based data breaches. This will enable future researchers and practitioners to utilize the dataset ethically without being concerned with real-life PIIs. This paper describes the synthetic datasets that are being generated for fictitious data breach scenarios, highlighting the PIIs distributed across 16 datasets using the Master Record Table (MRT) consisting of a full PII profile of 4 million individuals. The nature of the PIIs (data classes) is sourced from ‘Have I Been Pwned?’ (HIBP) [1]. All the records are synthetically generated. Taking cues from NIST SP 1800-29 [2], which provides guidance around the threat of data breaches, and ISO/IEC 27005:2022 [3], which provides information security risk management guidelines. These industry-standard documents enabled us to create fictitious scenarios for data breach datasets.

## 3 Data Description

This section describes the synthetically generated datasets. The datasets are of two types: Master Record Table (MRT) consisting of 1 dataset (description provided in Section 2.1) and Scenario-based data breach records which consists of 16 datasets (description provided in Sections 2.2 – 2.17). The MRT consists of a compilation of 4 million records of individuals consisting common PIIs sourced from HIBP. Furthemore, The 16 scenario-based datasets are derived from MRT and the narrative of the scenarios was created by taking cues from NIST SP 1800-29 and ISO/IEC 27005:2022. The narrative of the data breach scenarios is based around the year 2023.

### 3.1 Master record table (MRT)

We have synthetically generated 4 million records of unique individuals by creating their full profile (Table 1). The full profile of the individuals consists of the PII data classes sourced from HIBP. The MRT consists of the following PII labels – *‘Name’, ‘Phone Number’, ‘Email Address’, ‘DOB’, ‘Physical Address’, ‘City’, ‘State’, ‘Zip Code’, ‘Credit Card Number’, ‘CVV’, ‘Credit Card Expiry’, ‘Education Level’, ‘Device Information (OS + Version)’, ‘Username1’, ‘Username2’, ‘Government Issued IDs’, ‘Passwords1’, ‘Passwords2’*. MRT was generated so as to create scenario-based datasets. This will also serve as a demonstration on how the MRT can be used further by researchers and industry professionals to create new sets of records as per their requirements. Following are the details of the PII labels for the records that are synthetically generated:

• *Name*: Consists of unique names that are synthetically generated.
• *Phone Number*: Consists of USA based phone numbers, with international dialing code (+1), followed by a 10-digit number comprising of three-digit area code and a seven-digit local number.
• *Email Address*: Email addresses are mapped to the names that are synthetically generated.
• *DOB*: Consists of Date of birth for each individual. The date of birth ranges between years 1960 to 2002.
• *Physical Address*: Synthetically generated random house number and street name.
• *City*: Name of the city in USA
• *State*: Name of state in USA
• *Zip Code*: Zip code in USA based on the city and the state
• *Credit Card Number*: Uniquely generated 16 digit card number
• *CVV*: Consists of CVV (Card Verification Value) number
• *Credit Card Expiry*: Consists of card expiry date. The expiry dates are centered around 2016 to 2022.
• *Education Level*: Education levels of individuals. The education levels vary from the defined categories – “Less than High School”, “High School Graduate”, “Diploma”, “Bachelor’s Degree”, “Master’s Degree” and “Doctorate”.
• *Device Information (OS + Version)*: Consists details on the phone operating system details, either iOS or Android (versions released between 2016 and 2022)
• *username1*: List of usernames generated based on the unique names generated.
• *username2*: List of another set of usernames mapped to the unique names but it also consists of the numeric values appended to the usernames to create another variation in the username.
• *Government Issued IDs*: Type of government issued IDs for each individual, the predefined IDs are categorized as “Social Security Number”, “Driver’s License”, “State Identification Card” and “Passport”.

While the PII data classes listed above are synthetically generated, we also utilized the password list (*‘rockyou.txt’*) used by industry professionals and researchers in the area of cybersecurity.

• *passwords1*: List of unique passwords mapped to each individual sourced from ‘*rockyou.txt*’ password list.
• *passwords2*: Another list of passwords mapped to each individual sourced from ‘*rockyou.txt*’ password list.

**Table 1:** Master table record (MRT) dataset description.

Synthetic Dataset #	Data Classes Considered for the dataset
*Dataset MRT: Master Record Table* (4 Million Records)	Name, Phone Number, Email Address, DOB, Physical Address, City, State, Zip Code, Credit Card Number, CVV, Credit Card Expiry, Education Level, Device Information (OS + Version), passwords1, passwords2, username1, username2, Government Issued IDs

Based on the MRT, we created 16 datasets based on fictitious data breach scenarios, the data breach scenarios are based around the year 2023. The narrative of the events are based on the datasets that were leaked by the hacker group or individual(s) across several industries on the darkweb or the clear web.

### 3.2 Data breach scenario 1: banking app data

In February 2023, a hacker group on the dark web gained unauthorized access to a banking app’s backend and exfiltrated 3.2 million customer records with the PII data classes described in Table 2. Despite the bank's data being encrypted, weaknesses in the encryption protocol and API vulnerabilities allowed the hacker group to decrypt and expose sensitive customer information. The data was then dumped on the darknet hacking forums for sale.

**Table 2:** Description of the ‘Data Breach Scenario 1’ dataset.

Scenario-based Synthetic Dataset	PII Data Class labels of the dataset
*Data Breach Scenario 1: Banking App Data* (3.2 Million records)	1. ‘*Email Address’, ‘username1’, ‘Phone Number’, ‘passwords2’, ‘Name’, ‘Physical Addresses’, ‘City’, ‘State’, ‘Zip Code’, ‘Credit Card Number’, ‘CVV’, ‘Credit Card Expiry’, ‘Government Issued IDs’* – Consists of the records for each individual, all sampled and sourced from MRT. 2. ‘*Payment History’* – Consists of the records of the payment details by the individual.

### 3.3 Data breach scenario 2: online grocery shopping cart

This is a case of SQL Injection Attack. In June 2023, hackers exploited a vulnerability in the online grocery shopping cart's website by injecting malicious SQL code into user input fields. This allowed them to bypass security mechanisms and gain unauthorized access to the backend database, where they extracted 3 million customer records. The PII data classes are listed in Table 3. The stolen data was then used to make fraudulent purchases and the data was also sold on the dark web.

**Table 3:** Description of the ‘Data Breach Scenario 2’ dataset.

Scenario-based Synthetic Dataset	PII Data Class labels of the dataset
*Data Breach Scenario 2: Online Grocery Shopping Cart* (3 Million records)	1. *‘Phone Number’, ‘Name’, ‘Physical Address’, ‘City’, ‘State’, ‘Zip Code’, ‘password2’* – Consists of the records for each individual, all sampled and sourced from MRT. 2. *‘Payment History’* – Consists of the records of the payment details by the individual. 3. *‘Partial Credit Card Data’* – Consists of the last 4 digits of the card number and the CVV together, this is sourced from MRT. 4. *‘Browser User Agent’* – Consists of the browser user agent details of the individual.

### 3.4 Data breach scenario 3: online restaurant booking and reservation

In the late of May 2023, an online hacking group targeted a restaurant booking platform. This platform was vulnerable to a SQL injection attack, allowing hackers to access the database and extract 1.9 million customer records. The PII data classes are listed in Table 4. The stolen data was then used to carry out fraudulent transactions and identity theft. The data was further dumped on the clear web and the dark net forums.

**Table 4:** Description of the ‘Data Breach Scenario 3’ dataset.

Scenario-based Synthetic Dataset	PII Data Class labels of the dataset
*Data Breach Scenario 3: Online Restaurant Booking and Reservation* (1.9 Million records)	1. *‘Username1’, ‘Passwords1’, ‘Name’, ‘Phone Number’, ‘City’, ‘Zip Code’* – Consists of the records for each individual, all sampled and sourced from MRT. 2. *‘Payment Methods’* – Consists of the payment methods used by the individuals which can be the combination of any of these categories – Credit Card, Debit Card, Cash, Mobile Payment, Bank Transfer, Online Wallet 3. *‘Credit Card Number (Last 4 Digits)’* – Consists of the last 4 digits of the credit card, the data is sourced from MRT.

### 3.5 Data breach scenario 4: online learning platform

In early August 2023, a disgruntled employee with elevated access privileges misused his position to exfiltrate 1.9 million user records consisting of PII described in Table 5. This data was first sold in the hacking forum on the dark web and in the first week of September 2023, then the data was further dumped on the clear web which caused reputational damage to the organization.

**Table 5:** Description of the ‘Data Breach Scenario 4’ dataset.

Scenario-based Synthetic Dataset	PII Data Class labels of the dataset
*Data Breach Scenario 4: Online Learning Platform* (960,000 records)	1. *‘username2’, ‘Email Address’, ‘passwords1’, ‘Name’, ‘Education Level’* – Consists of the records for each individual, all sampled and sourced from MRT. 2. *‘Auth Token’* – Consists of the authentication token for each individual user for logging in to their accounts.

### 3.6 Data breach scenario 5: hotel booking

In June 2023, hackers breached a third-party vendor that processes payments for the hotel booking system. By exploiting this supply chain vulnerability, attackers gained access to 2.9 million user records with the compromised PIIs described in Table 6. The data was sold on underground forums and used for fraudulent transactions.

**Table 6:** Description of the ‘Data Breach Scenario 5’ dataset.

Scenario-based Synthetic Dataset	PII Data Class labels of the dataset
*Data Breach Scenario 5: Hotel Booking* (2.9 Million records)	1. *‘Username1’, ‘Email Address’, ‘passwords1’, ‘Name’, ‘Physical Address’, ‘City’, ‘Zip Code’, ‘Card Expiry Date’, ‘Device Information (OS + Version)’* – Consists of the records for each individual, all sampled and sourced from MRT. 2. *‘Payment Methods’* – Consists of the payment methods used by the individuals which can be the combination of any of these categories – “Credit Card”, “Debit Card”, “Mobile Payment”, “Cash”, “Bank Transfer" 3. *‘Travel Habits’* – Consists of the travel habits of the individuals, and is used in combination with any of the following – “Domestic Flights”, “International Flights”, “Train Travel”, “Road Trips”, “Cruises”. 4. *‘Card Last 4 digits’* - Consists of the last 4 digits of the credit card, the data is sourced from MRT.

### 3.7 Data breach scenario 6: professional networking platform

In mid November 2023, hackers exploited a vulnerable search function on the professional networking platform by injecting malicious SQL queries into input fields. This allowed them to access the backend database, where they retrieved sensitive user data which is described in Table 7. With this data, the attackers leaked a compiled list of 3.1 million users and sold their information on the dark web which was used for identity theft and scams involving fraudulent job offers.

**Table 7:** Description of the ‘Data Breach Scenario 6’ dataset.

Scenario-based Synthetic Dataset	PII Data Class labels of the dataset
*Data Breach Scenario 6: Professional Networking App* (3.1 Million records)	1. *‘Email Address’, ‘passwords2’, ‘Name’, ‘Education Level’* – Consists of the records for each individual, all sampled and sourced from MRT.

### 3.8 Data breach scenario 7: dating app

In mid February 2023, a hacker group infiltrated the dating app's servers and gained access to the entire user database, which includes sensitive information described in Table 8. The attackers exfiltrated the data, and instead of immediately selling it on the dark web, they publicly threatened to expose the identities of users unless the company pays a ransom. When the ransom demand was not met by the organization, the attackers released the full dataset of 2.4 million user records, causing widespread damage to the app's reputation and leading to public shaming, extortion attempts, and personal distress for the affected users. The leak included sensitive user preferences, which led to sparking legal actions and severe public scrutiny.

**Table 8:** Description of the ‘Data Breach Scenario 7’ dataset.

Scenario-based Synthetic Dataset	PII Data Class labels of the dataset
*Data Breach Scenario 7: Dating App* (2.4 Million records)	1. *‘Username2’, ‘Passwords2’, ‘Names’, ‘DOB’, ‘Education Levels’* – Consists of the records for each individual, all sampled and sourced from MRT. 2. *‘Relationship Status’* - Depicts the relationship status which can be any one of the following – “Single”, “Married”, “In a Relationship”, “Divorced”, “Widowed”. 3. *‘Exercising Habits’* – Consists of the habits which can be any one of the following: “No Exercise”, “Light Exercise”, “Moderate Exercise”, “Heavy Exercise”. 4. *‘Smoking Habits’* – Consists of the habits which can be any one of the following: “Non-Smoker”, “Occasional Smoker”, “Regular Smoker”. 5. *‘Drinking Habits’* – Consists of the habits which can be any one of the following: “Non-Drinker”, “Occasional Drinker”, “Regular Drinker”.

### 3.9 Data breach scenario 8: food delivery app

In June 2023, hackers compromised a third-party payment processing vendor used by the food delivery app to handle transactions. The attackers exploited a vulnerability in the vendor's systems, allowing them to access sensitive information of 1.8 million user accounts, including credit card data, addresses, order histories and other PIIs listed in Table 9. Since the food delivery app relied on this third-party service for all payments, the breach affected a significant portion of its customer base. The stolen data was sold on the dark web, leading to fraudulent transactions and identity theft.

**Table 9:** Description of the ‘Data Breach Scenario 8’ dataset.

Scenario-based Synthetic Dataset	PII Data Class labels of the dataset
*Data Breach Scenario 8: Food Delivery App* (1.8 Million Records)	1. *‘Email addresses’, ‘Phone Number’, ‘Passwords1’, ‘Names’, ‘Physical addresses’, ‘City’, ‘State’, ‘Zip Codes’, ‘CVV’, ‘Device Information’* – Consists of the records for each individual, all sampled and sourced from MRT. 2. *‘Payment Methods’* – Consists of the payment methods used by the individuals which can be the combination of any of these categories – Credit Card, Debit Card, Mobile Payment, Cash, Bank Transfer. 3. *‘Last Order’* – Consists of the payment detail of the last order. 4. *‘Credit Card Data’* – Consists of the last 4 digits of the credit card, the data is sourced from MRT.

### 3.10 Data breach scenario 9: online E-Commerce shopping

In June 2023, hackers exploited a vulnerability in the online e-commerce platform by injecting malicious SQL code. This allowed them to gain unauthorized access to the backend database, where they extracted 1.7 million customer records with PIIs compromised such as names, physical addresses, email addresses, and payment method as listed in Table 10. The attackers exfiltrated this data and used it to commit identity theft and fraud, while also selling the stolen data on the dark web. The breach caused significant financial and reputational damage to the platform.

**Table 10:** Description of the ‘Data Breach Scenario 9’ dataset.

Scenario-based Synthetic Dataset	PII Data Class labels of the dataset
*Data Breach Scenario 9: Online E-commerce Shopping* (1.7 Million records)	1. *‘Email addresses’, ‘Passwords2’, ‘Names’, ‘Physical addresses’, ‘city’, ‘state’, ‘Zip Codes’, ‘Card Number’, ‘CVV’, ‘Expiry Date’* - Consists of the records for each individual, all sampled and sourced from MRT. 2. *‘Browser User Agent’* – Consists of the browser user agent details of the individual. 3. *‘Payment Methods’* – Consists of the payment methods used by the individuals which can be the combination of any of these categories – “Credit Card”, “Debit Card”, “Mobile Payment”, “Cash”, “Bank Transfer”.

### 3.11 Data breach scenario 10: online pharmacy app

An employee with access to 2.4 million customers’ medical records and payment information used his position to extract sensitive PIIs listed in Table 11, which is later sold on the darknet market. The breach includes names, addresses, and credit card details, raising concerns about both financial fraud and data privacy.

**Table 11:** Description of the ‘Data Breach Scenario 10’.

Scenario-based Synthetic Dataset	PII Data Class labels of the dataset
*Data Breach Scenario 10: Online Pharmacy App* (2.4 Million records)	1. *‘Email addresses’, ‘Passwords1’, ‘Names’, ‘Physical addresses’, ‘State’, ‘City’, ‘Zip Codes’, ‘Card Number’, ‘CVV’, ‘Expiry Date’, ‘Government issued IDs’* - Consists of the records for each individual, all sampled and sourced from MRT. 2. *‘Payment Methods’* – Consists of the payment methods used by the individuals which can be the combination of any of these categories – “Credit Card”, “Debit Card”, “Mobile Payment”, “Cash”, “Bank Transfer”.

### 3.12 Data breach scenario 11: online flight booking

In April 2023, a group of cyber attackers launched a spear-phishing campaign targeting employees of the flight booking platform. Using stolen credentials, they accessed 2.8 million user accounts, extracting personal details, payment data, and travel preferences. The stolen information was then used to commit identity theft and financial fraud. The PIIs exposed are listed in Table 12.

**Table 12:** Description of the ‘Data Breach Scenario 11’ dataset.

Scenario-based Synthetic Dataset	PII Data Class labels of the dataset
*Data Breach Scenario 11: Online Flight Booking* (2.8 Million records)	1. *‘Email addresses’, ‘passwords1’, ‘Names’, ‘Physical addresses’, ‘City’, ‘Zip Code’, ‘Card Number’, ‘CVV’, ‘Expiry Date’, ‘Device information’* - Consists of the records for each individual, all sampled and sourced from MRT. 2. *‘Travel Habits’* – The travel habits are taken from the predefined categories: “Domestic Flights”, “International Flights”, “Train Travel”, “Road Trips”, “Cruises”, “No Travel"

### 3.13 Data breach scenario 12: fitness app

In October 2023, a dark web hacker group discovered a vulnerability in the fitness app's API, which was insufficiently secured and improperly authenticated. By exploiting this exposed API, the attackers were able to bypass security controls and directly access the app's backend systems. This allowed them to retrieve sensitive user information (1.1 million records), including usernames, email addresses and device information (Table 13). The stolen data was then dumped on the clear web, resulting in targeted phishing attacks, identity theft, and unauthorized access to users’ accounts. The breach exposed the app's lack of proper API authentication and authorization mechanisms.

**Table 13:** Description of the ‘Data Breach Scenario 12’ dataset.

Scenario-based Synthetic Dataset	PII Data Class labels of the dataset
*Data Breach Scenario 12: Fitness App* (1.1 Million records)	1. *‘Username2’, ‘Email addresses’, ‘Names’, ‘Device information’* – Consists of the records for each individual, all sampled and sourced from MRT.

### 3.14 Data breach scenario 13: cab booking

In late December 2023, attackers first execute a DDoS attack to overwhelm the cab booking service's servers, creating an opportunity for data exfiltration during the chaos. They successfully extract the customer payment methods and personal information, such as names, addresses, card details and other PIIs listed in Table 14. The Cab Booking company was first demanded a ransom and were given a deadline for the payment. Due to the non compliance of the ransom, the hacker group dumped the records of 2.1 million users on the clear web and the dark web. This caused damage to the reputation of the company and loss of customers.

**Table 14:** Description of the ‘Data Breach Scenario 13’ dataset.

Scenario-based Synthetic Dataset	PII Data Class labels of the dataset
*Data Breach Scenario 13: Cab Booking* (2.1 Million records)	1. *‘Email addresses’, ‘Passwords2’, ‘Names’, ‘Physical addresses’, ‘cvv’, ‘Card Expiry Date’, ‘Device information’, ‘City’, ‘Zip Code’* – Consists of the records for each individual, all sampled and sourced from MRT. 2. *‘Payment Methods’* – Consists of the payment methods used by the individuals which can be the combination of any of these categories – “Credit Card”, “Debit Card”, “Mobile Payment”, “Cash”, “Bank Transfer”. 3. *‘Card Last 4 digits’* – Consists of the last 4 digits of the credit card, the data is sourced from MRT.

### 3.15 Data breach scenario 14: movie booking

In March 2023, a disgruntled employee with elevated access privileges within the movie booking platform misused his access to extract sensitive users’ information, including names, email addresses, credit card details, and movie preferences (Table 15). The employee exported the data without authorization and sold it on the dark web. The breach exposed weak internal controls and a lack of monitoring for privileged access within the company. As a result, affected users faced identity theft and were impacted by fraudulent transactions.

**Table 15:** Description of the ‘Data Breach Scenario 14’ dataset.

Scenario-based Synthetic Dataset	PII Data Class labels of the dataset
*Data Breach Scenario 14: Movie Booking* (1.3 Million records)	1. *‘Username1’, ‘Passwords2’, ‘Names’, ‘Physical addresses’, ‘City’, ‘Zip Code’, ‘Card Number’, ‘cvv’, ‘Expiry Date’, ‘Device information’* – Consists of the records for each individual, all sampled and sourced from MRT. 2. *‘Movie Preferences’* – Consists of random combinations of the movie preferences form the following categories: “Action”, “Comedy”, “Drama”, “Horror”, “Science Fiction”, “Romance”, “Documentary”, “Thriller"

### 3.16 Data breach scenario 15: video editing app

In February 2023, a hacker group created a fake app store which consisted of information stealing malware embedded into the video editing app, which was downloaded by 800,000 users. After gaining unauthorized access to user accounts, they extracted email addresses and device information (Table 16) which was then leaked online.

**Table 16:** Description of the ‘Data Breach Scenario 15’ dataset.

Scenario-based Synthetic Dataset	PII Data Class labels of the dataset
*Data Breach Scenario 15: Video Editing App* (800,000 records)	1. *‘Email addresses’, ‘Device information’* – Consists of the records for each individual, all sampled and sourced from MRT.

### 3.17 Data breach scenario 16: study time table app

In July 2023, a hacker group used previously stolen usernames and passwords from other breaches to perform credential stuffing attacks on a study planning and timetable app. The compromised accounts expose personal educational details, email addresses, device information along with app usage behaviour listed in Table 17. The attackers used this data to create fake profiles and also dumped the information of 600,000 compromised user accounts on the clear web.

**Table 17:** Description of the ‘Data Breach Scenario 16’ dataset.

Scenario-based Synthetic Dataset	PII Data Class labels of the dataset
*Data Breach Scenario 16: Study Time Table App* (600,000 records)	1. *‘Email addresses’, ‘Passwords1’, ‘Names’, ‘Education level’, ‘Device information’* – Consists of the records for each individual, all sampled and sourced from MRT. 2. *‘App Usage Data’* – Consists of usage behaviour based on the following categories: “hourly for study sessions”, “daily for planning”, “weekly for reviews", this reflects users’ app engagement behaviors

## 4 Experimental Design, Materials and Methods

### 4.1 Generating master record table (MRT)

**Fig. 1:**
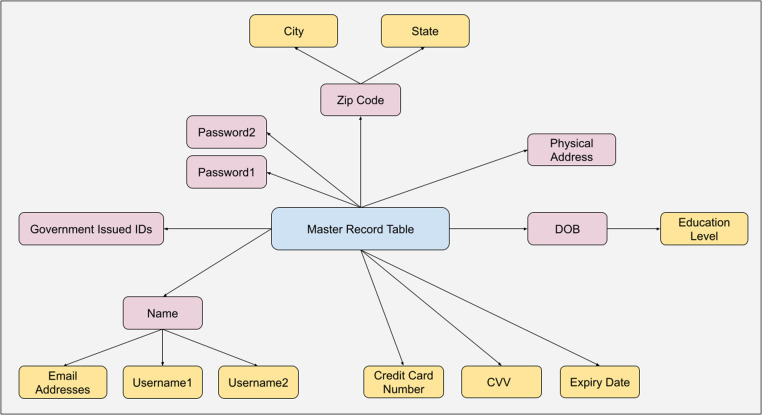
Overview of the synthetic data generation of MRT (Legend: Blue – main aggregated source; purple – first level records; orange – second level records that are derived from the first level records).

The Master Record Table (MRT) is a synthetically generated dataset containing complete profiles of 4 million unique individuals, designed to represent a wide variety of PII (Table 1). This dataset is the foundation for generating scenario-based data breach records. The data generation process was structured in two levels, as illustrated in Fig. 1, which shows how first-level records (e.g., names, physical addresses, zip code) were generated and then used to derive second-level records (e.g., city, state, usernames, email addresses). The following is a description of the steps taken to create the MRT:

Step 1: The process began with the generation of 4 million unique names (*‘Name’*) using the Faker Python library. Based on these names, corresponding email addresses (‘Email Address’) were created to mimic realistic email patterns based on names along with the synthetic domain names. Additionally, two unique usernames were generated for each individual. The first username list (‘*username1’*) was created from the individual’s name, using a combination of their first and last names, while the second username list (‘*username2’*) was a variation of the first but random numbers were appended at the end to mimic the common real-world practice of users having multiple usernames across different platforms.

Step 2: We then programmatically generated the date of birth (‘*DOB’*) for each individual, ensuring that all individuals were over 18 years old as of the fictitious breach year (2023). The DOBs were generated for individuals born between 1960 and 2002. Based on their DOB, we assigned education levels (‘*Education Level’*) mimicking realistic probabilities that reflect educational level patterns across different age groups. For this, individuals who were aged 18–24 were assigned a “High School Graduate” or “Diploma,” while older individuals, who were in the 25–34 age range, were assigned “Bachelor's,” “Master's,” or even “Doctorate” degrees. Thus following were the education levels assigned across: “Less than High School”, “High School Graduate”, “Diploma”, “Bachelor’s Degree”, “Master’s Degree”, “Doctorate”.

Step 3: Next, we generated synthetic physical addresses, including house numbers and street names, using Faker. These were combined with city (*‘City’*), state (*‘State’*), and zip codes (*‘Zip Code’*) generated by the ‘*pyzipcode*’ library. We were able to create unique address combinations for each individual, reflecting the diverse geographic locations.

Step 4: We further synthetically generated credit card records (*‘Credit Card Number’*). We generated a unique 16-digit credit card numbers programmatically, along with the corresponding CVV (*‘CVV’*) and expiry date of the card (*‘Credit Card Expiry’*). The expiry dates was hardcoded around years 2016 to 2022.

Step 5: In this record, each individual in the record was assigned a phone number (*‘Phone Number’*) following the format used in the United States. The numbers included an international dialing code (+1), followed by a 10-digit number comprising of three-digit area code and a seven-digit local number. Also, for the generated phone records, we excluded real-world area codes (taken from North American Numbering Plan Administrator – NANPA). This ensured that the phone numbers generated are synthetic in nature.

Step 6: In this step we generated list of passwords which were sourced from ‘rockyou.txt’ password lists. We assigned two unique password lists (‘*passwords1’* and ‘*passwords2’* ). The passwords randomly selected from the ‘rockyou.txt’ list and there was no duplicates across the two new lists that we have generated.

Step 7: For device information (‘*Device Information (OS + Version)*’), we generated details about the operating system (either iOS or Android) and the version number, selecting versions released between 2016 and 2022.

Step 8: Lastly, we assigned each individual one or more randomly selected government-issued IDs (‘*Government Issued IDs’*), such as Social Security Numbers (SSN), Driver’s Licenses, State Identification Cards, and Passport.

### 4.2 Generating datasets sourced from MRT

The Master Record Table (MRT) was used for generating 16 scenario-based data breach datasets, each dataset represents different industry type and data breach incident. These 16 datasets were created by selecting relevant PIIs from the MRT and also mixing it with additional field(s) specific to each scenario. Below is a summary of how each dataset was generated:

1. *‘Data Breach Scenario 1: Banking App Data’* – To generate dataset for this scenario, the following PII data classes were taken from the MRT: ‘Email Address’, ‘Username1’, ‘Phone Number’, ‘Passwords2’, ‘Name’, ‘Physical Address’, ‘City’, ‘State’, ‘Zip Code’, ‘Credit Card Number’, ‘CVV’, ‘Credit Card Expiry’, and ‘Government Issued IDs’ (Table 2). In order to simulate in a banking context ‘Payment History’ label was added intentionally which included five random payments. These were created for each individual, including details such as payment method, amount, recipient, and dates centered around a specific range. This added record label mimicks real-life transaction behavior.
2. *‘Data Breach Scenario 2: Online Grocery Shopping Cart*’ – To generate dataset for this scenario, the following PII data classes were selected from the MRT: ‘Phone Number’, ‘Name’, ‘Physical Address’, ‘City’, ‘State’, ‘Zip Code’, ‘Passwords2’, and ‘Partial Credit Card Data’ (last 4 digits of the card number) (Table 3). Additional fields such as ‘Payment History’ and ‘Browser User Agent’ were added. Purchase history records were randomly generated, including payment methods and transaction amounts, to mimic some recent purchases. The Browser User Agent was randomly generated using the ‘fake-useragent’ Python library, which can reveal what type of browser an individual is using.
3. *‘Data Breach Scenario 3: Online Restaurant Booking and Reservation’* – To generate dataset for this scenario, the following PII data classes were sourced from the MRT: ‘Username1’, ‘Passwords1’, ‘Name’, ‘Phone Number’, ‘City’, ‘Zip Code’, and ‘Credit Card Number (Last 4 Digits)’ (Table 4). To this list of PIIs, we added a ‘Payment Methods’ field that included categories: Credit Card, Debit Card, Cash, Mobile Payment, Bank Transfer, Online Wallet. These categories were used to assign various payment options the online users’ were using on the restaurant booking platform.
4. *‘Data Breach Scenario 4: Online Learning Platform’* – To generate a dataset for this scenario, the following PII data classes were selected from the MRT: ‘Username2’, ‘Email Address’, ‘Passwords1’, ‘Name’, and ‘Education Level’ (Table 5). For this dataset scenario, an added ‘Auth Token’ label for each individual which represented the authentication tokens used in the online learning platform for user authentication. Using the Faker library, a unique, random 64-character hash string was generated.
5. *‘Data Breach Scenario 5: Hotel Booking’* – To generate dataset for this scenario, the following PII data classes were selected from the MRT: ‘Username1’, ‘Email Address’, ‘Passwords1’, ‘Name’, ‘Physical Address’, ‘City’, ‘Zip Code’, ‘Credit Card Expiry’, ‘Card Last 4 digits’ and ‘Device Information’ (Table 6). Additional fields included ‘Payment Methods’ and ‘Travel Habits’. For each individuals, travel preferences (“Domestic Flights”, “International Flights”, “Train Travel”, “Road Trips”, “Cruises”) were randomly selected in a combination. This mimicked diverse travel behaviors typical of hotel users.
6. *‘Data Breach Scenario 6: Professional Networking Platform*’ – To generate dataset for this scenario, the following PII data classes were taken from MRT: ‘Email Address’, ‘Passwords2’, ‘Name’, and ‘Education Level’ (Table 7). No additional fields were added.
7. *‘Data Breach Scenario 7: Dating App’* – To generate dataset for this scenario, the following PII data classes were selected from the MRT: ‘Username2’, ‘Passwords2’, ‘Names’, ‘DOB’, and ‘Education Level’ (Table 8). Additional fields such as ‘Relationship Status’ (with categories: “Single”, “Married”, “In a Relationship”, “Divorced”, “Widowed”), ‘Exercising Habits’ (with categories: “No Exercise”, “Light Exercise”, “Moderate Exercise”, “Heavy Exercise”), ‘Smoking Habits’ (with categories: “Non-Smoker”, “Occasional Smoker”, “Regular Smoker”), and ‘Drinking Habits’ (with categories: “Non-Drinker”, “Occasional Drinker”, “Regular Drinker”) were randomly assigned to each individual.
8. *‘Data Breach Scenario 8: Food Delivery App’* – To generate dataset for this scenario, PII data classes selected from the MRT included ‘Email Address’, ‘Phone Number’, ‘Passwords1’, ‘Name’, ‘Physical Address’, ‘City’, ‘State’, ‘Zip Code’, ‘CVV’, ‘Credit Card Data’ and ‘Device Information’ (Table 9). Additional fields such as ‘Payment Methods’ and ‘Last Order’ was generated to simulate food delivery app behavior. To the ‘Last Order’ record, we added a random value for amounts ranging between 8 USD to 120 USD to mimic purchase history in the app database.
9. ‘*Data Breach Scenario 9: Online E-Commerce Shopping’* – To generate a dataset for this scenario, the following PII data classes were selected from the MRT: ‘Email Address’, ‘Passwords2’, ‘Name’, ‘Physical Address’, ‘City’, ‘State’, ‘Zip Codes’, ‘Credit Card Number’, ‘CVV’, and ‘Expiry Date’ (Table 10). Additional fields such as ‘Browser User Agent’ and ‘Payment Methods’ were generated to simulate user shopping habits.
10. ‘*Data Breach Scenario 10: Online Pharmacy App*’ – To generate dataset for this scenario, the following PII data classes were selected from the MRT: ‘Email Address’, ‘Passwords1’, ‘Name’, ‘Physical Address’, ‘State’, ‘City’, ‘Zip Code’, ‘Card Number’, ‘CVV’, ‘Expiry Date’, and ‘Government Issued IDs’ (Table 11). Like previous datasets ‘Payment Methods’ label was added to reflect pharmacy app users’ purchases.
11. ‘*Data Breach Scenario 11: Online Flight Booking’* – To generate dataset for this scenario, PII data classes sourced from the MRT included ‘Email Address’, ‘Passwords1’, ‘Name’, ‘Physical Address’, ‘City’, ‘Zip Code’, ‘Card Number’, ‘CVV’, ‘Expiry Date’, and ‘Device Information’ (Table 12). Additional label ‘Travel Habits’ (with categories: “Domestic Flights”, “International Flights”, “Train Travel”, “Road Trips”, “Cruises”, “No Travel”) was added. For this we assigned random travel preference selected by each user.
12. ‘*Data Breach Scenario 12: Fitness App’* – To generate a dataset for this scenario, the following PII data classes were selected from the MRT: ‘Username2’, ‘Email Address’, ‘Names’, and ‘Device Information’ (Table 13).
13. ‘*Data Breach Scenario 13: Cab Booking*’ – To generate dataset for this scenario, PII data classes sourced from the MRT included ‘Email Address’, ‘Passwords2’, ‘Name’, ‘Physical Address’, ‘CVV’, ‘Card Expiry Date’, ‘Device Information’, ‘City’, ‘Zip Code’, and ‘Card Last 4 Digits’ (Table 14). We also added ‘Payment Methods’ label, simulating typical cab booking payment methods.
14. *‘Data Breach Scenario 14: Movie Booking*’ – To generate dataset for this scenario, the following PII data classes were selected from the MRT: ‘Username1’, ‘Passwords2’, ‘Names’, ‘Physical Address’, ‘City’, ‘Zip Code’, ‘Card Number’, ‘CVV’, ‘Expiry Date’, and ‘Device Information’ (Table 15). We further added a ‘Movie Preferences’ label (with categories: “Action”, “Comedy”, “Drama”, “Horror”, “Science Fiction”, “Romance”, “Documentary”, “Thriller”), reflecting the variety of user preferences in a movie booking platform.
15. *‘Data Breach Scenario 15: Video Editing App’* – To generate a dataset for this scenario, the following PII data classes were selected from the MRT: ‘Email Addresses’ and ‘Device Information’ (Table 16).
16. *‘Data Breach Scenario 16: Study Time Table App’* – To generate dataset for this scenario, PII data classes sourced from the MRT included ‘Email Addresses’, ‘Passwords1’, ‘Names’, ‘Education Level’, and ‘Device Information’ (Table 17). We further added ‘App Usage Data’ label (with the categories: “hourly for study sessions”, “daily for planning”, “weekly for reviews”) reflecting users’ app engagement behaviors.

The generated datasets provided with the paper, which exceed 500 MB in file size, have been split into smaller, scenario-specific files to facilitate easier handling and better understanding. These split datasets are stored alongside the original dataset to offer flexibility and accessibility for researchers and industry practitioners. The original and split datasets are provided in ‘*.csv*’ format, ensuring compatibility with common data analysis libraries (such as ‘*pandas*’) and other widely used analytical tools. Detailed instructions and short code examples for loading, splitting, data generation, and working with the datasets are included as part of the dataset package. The workings of the code are further explained in comments within the code files and an accompanying introductory ‘*README.txt’* file.

## Limitations

There are a few limitations in the datasets. Some biases were included in the generation of the datasets. First, the dataset consists of addresses (city, state, zip code) and phone numbers based in the United States. This was done so as not to overcomplicate or overload the dataset with information from different geographic regions that might lead to complications in datasets. Second, we have not considered either assigning gender or the GPS locations. Gender can be a sensitive and personal attribute that we exclude for oversimplification or misrepresentation. Also, the dataset primarily focused on attributes such as names, phone numbers, email addresses, dates of birth, addresses, states, credit card details, and other data classes that are more relevant to real-life hacked data. On the other hand, GPS coordinates were not included so as to obfuscate the details, so that it is not linked randomly to some addresses on the geographical maps that might lead to some concerns, rather we used synthetically generated street names and house numbers.

## Ethics Statement

This study involves the generation of synthetic data to simulate data breach records. No human subjects were involved in this study, and no personal data was collected or used.

## Credit Author Statement

**Abhishek Sharma:** Conceptualization, Data Curation, Formal Analysis, Resources, Software, Writing – original draft, Writing – review and editing. **May Bantan:** Conceptualization, Supervision, Writing – review and editing.

## Declaration of Generative AI in Scientific Writing

During the preparation of this work the author(s) used ‘Grammarly’ and locally hosted (on device) Meta’s Large Language Model ‘Llama 3.1 8B’ in order to improve the readability, language and grammar correction. After using this tool/service, the author(s) reviewed and edited the content as needed and take(s) full responsibility for the content of the published article.

## Data Availability

Mendeley DataSynthetic Datasets for Scenario-based Data Breaches (Original data). Mendeley DataSynthetic Datasets for Scenario-based Data Breaches (Original data).
